# A new effective method in the treatment of severe ovarian hyperstimulation syndrome

**Published:** 2012-11

**Authors:** Qingrui Zhang, Liangbin Xia, Gao Gao

**Affiliations:** *Department of Gynecology, Renmin Hospital of Wuhan University, Wuhan, China.*

**Keywords:** *OHSS*, *Infertility*, *Ascites*, *Transfusion*

## Abstract

**Background: **Ovarian hyperstimulation syndrome (OHSS) is a recognized complication of ovulation induction, occurring in 1-10% of IVF and embryo transfer cycles. While mild OHSS is of no clinical relevance, severe OHSS is a life threatening complication. However, the efficacy of prevalent treatments appeared to be limited. We developed a continuous autotransfusion system with an ultrafiltration instrument for reinfusion the protein of concentrated ascites for the treatment of severe OHSS.

**Objective:** To study the efficacy and safety of using a continuous autotransfusion system for the treatment of severe OHSS.

**Materials and Methods:** 69 patients with severe OHSS who were treated with controlled ovarian hyperstimulation due to infertility from February 2002 to August 2010 in our reproductive center were divided into two groups. One group treated with continuous autotransfusion system with an ultrafiltration instrument which infused the protein of concentrated ascites, called ultrafiltration group, another group were treated with simple-albumin supplement, called albumin group. Several examinational results and adverse effect were compared between the two groups.

**Results:** The volume of urine output after 72h in ultrafiltration group was more than that in albumin group, the waist circumference and body weight in ultrafiltration group were lower than those in albumin group after 72h. The serum creatinine levels after 72h in ultrafiltration group was still significantly lower than that in albumin group (p<0.05). The ultrafiltration group rarely showed adverse effect compared with albumin group.

**Conclusion:** Autotransfusion of protein in concentrated ascites for the treatment of severe ovarian hyperstimulation syndrome was effective and safe.

## Introduction

Ovarian hyperstimulation syndrome (OHSS) is a recognized complication of ovulation induction, occurring in 1-10% of IVF and embryo transfer cycles (1). The syndrome is characterized by cystic enlargement of the ovaries and a fluid shift from the intravascular to the third space due to increased capillary permeability and ovarian neoangiogenesis. Its occurrence is dependent on the administration of human chorionic gonadotrophin (hCG) (2). 

While mild OHSS is of no clinical relevance, severe OHSS-characterized by massive ovarian enlargement, ascites, and pleural effusion, oliguria, haemoconcentration and thromboembolic phenomena-is a lifethreatening complication (3). 

However, its treatment has remained empirical (4). Intravenous fluid therapy using albumin solution, the use of heparin for coagulopathy, the use of dopamine to increase renal blood flow, paracentesis, peritoneovenous shunting and aspiration of follicular fluid have been reported for the management of severe OHSS (5-11). 

However, the efficacy of these treatments appeared to be limited. Moreover, albumin is scarce and expensive in China. Accordingly, we developed a continuous autotransfusion system with an ultrafiltration instrument for reinfusion the protein of concentrated ascites for the treatment of severe OHSS. Therefore we made use of the albumin from autotransfusion ascites without rejection and infection. The clinical dates were reported as follows.

## Materials and methods

**Research objects**

In this Historical cohort study 69 patients, from Department of Gynecology, Renmin Hospital of Wuhan University, Wuhan, China with severe OHSS were observed. They were treated with controlled ovarian hyperstimulation due to infertility from February 2002 to August 2010. All patients had infertility for 1-14 years including 31 cases of primary infertility, and 38 cases of secondary infertility. Their ages range from 25y to 39y. The diagnosis standard is in accord with reference literature (2). 30 cases were treated with continuous autotransfusion system with an ultrafiltration instrument which infused the protein of concentrated ascites, while the other 39 cases were treated with simple-albumin supplement. The criterion of this study is the diameter of ovary between 5-8cm, and with no complication. All patients and control subjects were approved by ethic committee of Wuhan University and had given written informed consent.

**Ovarian hyperstimulation protocol**

All the patients were using short protocol. 0.1mg triptorelin (produced by Ferring Hongkong Phamaceuticals) S.C. daily starting on day 2 of menstruation, from the first to third days of using triptorelin, the level of follicle-stimulation hormone (FSH) and luteinizing hormone (LH) rose, at the late period ,it turned to suppress the secretion of FSH and LH. 

Clomiphene is daily used in ovarian stimulation on the 3^rd^ day of menstruation, the ultrasound examination was performed to monitor the follicle growth status and blood was collected to test the level of estrogenic hormone. 10000IU HCG I.M. starting when oocyte matured judged by ultrasound to promote the maturation of oocyte. 36 hours after HCG injection, oocyte can be retrieved.

**Clinical manifestation**

The 69 patients presented different clinical symptoms, such as abdominal dropsy (69 cases); pleural fluid (24 cases); coeliodynia (16 cases); nausea and vomiting (25 cases); chest distress (18 cases); oliguresis (55 cases); and ovaries accretion (69 cases); All the clinical manifestation appeared after 5-12 days when patients were injected with ovulation stimulants.

**Treatment method**

Ultrafitration group: The FSCL2LY-A hydroperitoneum ultrafitration equipment was used. The consumable materials were hollow filament strain and polyethylene arterial and venous ducts. Ascites puncture was operated as routine to draw the ascites from the left lower quadrant at flow rate of 150ml/s to 250ml/s with barotropic pump. The middle molecule and micromolecule in ascites were filtrated when they went through strainer under negative pressure. 

The concentrated ascites was infused back to abdominal cavity transvenous catheter from the right upper quadrant. The treatment was finished until the hydroperitoneum couldn’t be educed persistently. The whole process was under sterile operation and in closed environment. About 800-1000ml filtrate was gained after one time of ultrafitration. The time of therapy lasted four hours. Abdomen was pressurized by abdominal bandage for 4 -6 hours after the operation to prevent the occurrence of shock by releasing ascites abruptly.

**Observation index**

All patients were routinely tested for Blood-Rt, Urine-Rt, liver and renal function, and electrolyte after hospital admission. The sizes of ovaries, the volume of pleural and peritoneal fluid were monitored by ultrasound at the right moment. The rational symptoms and complication after treatment and the adverse reaction which occurred during the theraputic process were observed every day. Urine volume, abdomen circumference, and body weight were recorded every day, while the development of some biochemical indicators, such as hematokrit, electrolyte, creatinine, albumin, and total protein were monitored abidingly. Venous blood from both groups were retaken to measure biochemical indicators 72 hours after treament. 

**Criterion of therapeutic effect**

Eased rational symptoms, palliated abdominal distension, more than 400ml urinary volume per day, a balanced fluid to go out and come in and a normal range of HCT were thought to be effective after Ultrafitration treatment.

**Statistical analysis**

Results are expressed as the mean± SD. In univariate model, t-test was used for continuous variables analysis and A Yates corrected x 2-test was used for discrete variables analysis. The biochemical parameters between the two groups were analyzed with the Student’s t-test. All data were processed by the statistical software (SPSS 11.0 for Windows; SPSS, Chicago, IL). p<0.05 was considered statistically significant.

## Results

**Comparison of general clinical features in two groups**

There were no significant differences of the two groups in age, body mass index (BMI), infertility duration, gravidity, parity and the interval between infusion of hCG and onset of OHSS. There were no significant differences in numbers of ovarian follicles and pregnancy rate either. In ultrafiltration group the time of hospital stay were much shorter than that in albumin group, and the difference was of statistical significance. Constituent ratio of patients who supplemented with heparin in ultrafiltration group was significantly higher than that in albumin group. Constituent ratio of patients who supplied with dopamine ultrafiltration group was slightly lower than that in albumin group, but the difference was of no significance. 

**Comparison of examinational results before and after treatment in two groups**

The volume of urine output increased remarkably after ultrafiltration. The volume of urine output after 72h in ultrafiltration group was more than that in albumin group. The waist circumference and body weight in ultrafiltration group were lower than those in albumin group after 72h. After ultrafiltration the serum potassium, sodium and chloridion concentration elevated, but was of no significance. The serum potassium, sodium and chloridion concentration after 72h in ultrafiltration group were somewhat higher than those in albumin group, which were in normal range and of no significance. 

A higher hematocrit (Ht) was detected after ultrafiltration, and Ht in ultrafiltration group after 72h was somewhat higher than Ht in albumin group, which was of no significance. After ultrafiltration the serum creatinine levels of patients declined markedly, and the serum creatinine levels after 72h in ultrafiltration group was still significantly lower than that in albumin group (Table II). No patients underwent repeated ultrafiltration treatment. Everyday cost of albumin group for each patient is 400 RMB (about 70USD).

**Gestational follow-up visit**

In the 69 cases of patients, 45 were pregnant (65.2%),and meanwhile 28 cases of patients had term delivery. two patients were in duration of pregnancy. 5 cases of patients were made a definite diagnosis of eccyesis, 7 cases had spontaneous abortion in early pregnancy and 3cases had premature delivery.

**Adverse effect**

The patients who were cured with ascites ultrafiltration equipment didn’t show adverse effect. Three in 30 patients presented fever (<38^o^C) within 24h after operation considering infections of abdominal cavity. Body temperature went back to normal after 7days of antibiotic treatment. Two patients presented abdominal pain which relieved soon after given symptomatic treatment. Six patients of albumin group presented fever, while two of them had fever reaching 38^o^C. When they were given symptomatic treatment of cooling, their body temperature went back to normal. No severe adverse effects were observed including shiver, hyperpyrexia, blood pressure decline, DIC, upper gastrointestinal bleeding etc.

**Table I:** Comparison of general characteristics of the two groups

	**Ultrafiltration group**	**Albumin group**	**p-value**
Age (years)	30.4 **± 2.4**	31.3 **± 3.6**	0.23
BMI	20.5 **± 1.6**	21.6 **± 2.1**	0.12
Duration of infertility (years)	6.2 **± 2.5**	7.5 **± 2.6**	0.31
Gravida and para	3.3 **± 1.1**	2.9 **± 1.4**	0.18
Interval between infusion of hCG and onset	7.6 **± 2.0**	6.4 **± 1.8**	0.25
No. of ovarian follicles	16.1 **± 1.3**	15.1 **± 1.5**	0.10
Pregnancy rate	21.5% **± 0.6%**	23.2% **± 0.4%**	0.21
Hospital stay (days)	5.8 **± 2.1**	9.3 **± 2.5**	0.03
Constituent ratio of heparin	**36.3%**	**17.6%**	0.02
Constituent ratio of dopamine	**33.7%**	35.1%	0.28

The data revised ‘16.1 ‘and ‘15.1’ above are the number of follicles in both ovaries, the original data’8.4’ and ‘7.8 ‘were follicles in one ovary.

**Table II:** Comparison of examinational results before and after treatment in two groups

**Items**	**Normal value**	Ultrafiltration group			Albumin group			**p**
		**Before ultrafiltration**	**After 72h**	**p**	**Before treatment**	**After 72h**	**p**	
Hematocrit (Ht) (%)	33.4-51.2	48.9**±7.4**	42.9**±6.7**	0.034	49.3**±6.7**	44.7**±5.0**	0.007	0.22
Total protein (g/L)	60-80	49.8**±13.2**	62.2**±11.5**	0.03	49.2**±12.3**	63.89**±7.32**	0.013	0.07
Albumin (g/L)	35-55	26.9**±10.2**	40.8**±12.6**	0.012	27.2**±10.0**	38.3**±9.7**	0.02	0.10
Creatinine (µmol/l)	54-133	157.23**±39.48**	126.82**±30.94**	0.043	155.42**±36.58**	149.44**±39.34**	ns	0.034
Urine output (ml/day)	1500-2500	1306**±160**	2079**±272**	0.012	1200**±142**	1612**±189**	0.045	0.031
Waist circumferences (cm)		95.15**±10.03**	69.15**±5.72**	0.022	93.24**±9.22**	80.87**±7.72**	0.039	0.029
Body weight (Kg)		79.1**±10.3**	**60.5±8.2**	**0.014**	81.8**±8.2**	71.9**±6.7**	**0.038**	**0.026**
Serum potassium (M)	3.5-5.5	3.96**±0.72**	4.22**±0.39**	0.17	3.88**±0.71**	4.15**±0.42**	0.09	0.17
Serum sodium (M)	135-145	132.31**±7.62**	134.25**±6.34**	0.09	131.65**±5.83**	133.13**±5.64**	0.12	0.08
Serum chlorine (M)	96-108	103.56**±7.86**	105.76**±6.75**	0.11	104.89**±8.06**	103.64**±7.31**	0.06	0.13

NS stands for p>0.05 and differences are of no significance.

## Discussion

OHSS is the major complication of ovarian stimulation and its most severe form can even threaten the patient’s life (12). Several strategies for preventing the syndrome have been described. Severe OHSS is a potentially fatal iatrogenic disease resulting in the accumulation of large amounts of ascites and pleural effusion, along with many other signs and symptoms (13). 

Conservative treatment of this condition includes hospitalization and correction of hypovolemia, hypoalbuminemia, and electrolyte imbalance by infusions of fluids, colloids and proteins (14). Additional drainage of ascitic fluid is necessary in patients with acute respiratory distress, with intractable pain caused by abdominal wall tension, and with oliguria. In these cases, paracentesis will alleviate pain, improve respiratory function, and increase diuresis (15). 

But a rapid re-accumulation of ascites and a decrease of the effective intravascular volume made the favorable effects limited. Exogenous albumin supplement is necessary because of the loss of protein in these cases. Toshimitsu *et al* reported a continuous autotransfusion system of ascites with a peritoneo-antecubital vein shunt for the treatment of severe OHSS (9). 

In these cases the ascites were reinfused into venous without concentration by ultrafiltration. It was questionable that the substances responsible for the syndrome, e.g. cytokines, re-entering the general circulation, may leading to exacerbation of OHSS.

FSCLZLY-A ascites ultrafiltration therapeutic apparatus (for short therapeutic apparatus), it is a special apparatus used to ultrafiltrate concentrate ascites and feedback it to abdominal cavity. The apparatus is made up of mainframe molecule screen filter pipeline and operating-bracket. The ascties is pumped by mainway-pump1, through center-empty fibre pipe, and effuses fitter, then renew feedback to abdominal cavity. Adjusting the fluxs of mainway-pump1 and branchway-pump2, and make the 1’s fluxs exceed to 2’s, mainway-pump pressure P1>branchway-pump pressure P2, then the branchway-pump pressure is negative, 

The water, cytokine in ascetic fluid is pumped through center-empty fibre pipe wall, and effuses, while protein renews feedback to abdominal cavity. The volume of the ascitic fluid aspirated ranged from 4000-19000 ml (mostly 6000~8000 ml), aspirated during 1.5~3 hours. The ultrafiltration and reinfusion system can remove cytokines and ascitic fluids without losing the protein. The advantage of this technique is that the patient’s own protein and electrolytes can be used. It was demonstrated that autotransfusion protein in concentrated ascites was successful in shortening hospital stay and reducing symptoms in patients with severe OHSS. Two cases were shortening hospital stay for ten days then other severe OHSS patients. In conclusion, the newly developed apparatus appeared to lead to the improvement of clinical symptoms, shortening hospital stay and alleviating burden for the patients of severe OHSS. 
